# Synthesis of an excellent MTP catalyst: hierarchical ZSM-5 zeolites with great mesoporosity

**DOI:** 10.1098/rsos.181691

**Published:** 2018-12-12

**Authors:** Guoqiang Song, Wenting Chen, Peipei Dang, Yuanyi Wang, Fuxiang Li

**Affiliations:** 1 School of chemical engineering, Guizhou Institute of Technology, 1st Caiguan Road, Yunyan District, Guiyang City 550003, Guizhou Province, People's Republic of China; 2 2011 Special Functional Materials Collaborative Innovation Center of Guizhou Province, Guizhou Institute of Technology, 1st Caiguan Road, Yunyan District, Guiyang City 550003, Guizhou Province, People's Republic of China; 3 Key Laboratory of Light Metal Materials Processing Technology of Guizhou Province, Guizhou Institute of Technology, 1st Caiguan Road, Yunyan District, Guiyang City 550003, Guizhou Province, People's Republic of China; 4 College of chemistry and chemical engineering, Taiyuan University of Technology, No. 79 Yingze west street, Taiyuan City 030024, Shanxi Province, People's Republic of China

**Keywords:** hierarchical, ZSM-5 zeolites, polyether amine, organosiloxane, methanol to propylene

## Abstract

A unique organosiloxane-polyether amine (OPA) was produced and used as mesoporogen to efficiently synthesize hierarchical ZSM-5 zeolites with great mesoporosity. We have employed silica sol and tetraethylorthosilicate, respectively, to investigate the influence of different silicon sources on hierarchical zeolites in the presence of OPA. The mesopores of synthesized samples focused on 6–15 nm, and the external surface area varied from 185 to 463 m^2^ g^−1^ where the micropore surface area was maintained at 245–334 m^2^ g^−1^. Benefiting from the superior structure properties, these samples were used as catalysts in the reaction of methanol to propylene, and the optimal one catalysed for 180 h with methanol conversion above 95%. The as-produced OPA could connect steadily with zeolite frameworks through covalent bonds (–Si–O–Si–) during the hydrothermal crystallization process. This type of connection mode could effectively avoid the formation of amorphous phase and the special molecular structure of OPA could efficiently introduce abundant mesopores with few micropores being consumed. The samples synthesized with silicon sol were made up of quasi-circular particles of about 800 nm in size and further consisted of nanocrystals of 40 nm, and the samples produced with TEOS have a particle size of about 1–2 µm aggregated with nanocrystals of 300 nm.

## 1. Introduction

In the petrochemical industry, propylene has been used as a feed stock to produce various polymer and chemical intermediates [1]. However, due to the great demand for propylene, a new process should be developed to supplement the increasing needs of propylene which was produced by the conventional method of petroleum cracking [2].

Because of the more accessible and economical nature of methanol [3], the process of methanol-to-propylene (MTP) developed by Lurgi has received great attention [4]. As an efficient route to produce propylene, the most crucial step of the MTP reaction was to optimize the production of olefins which was influenced greatly by the pore properties and the acidity of catalysts.

Based on the surface acidity, large surface area, excellent hydrothermal stability and the specific sieving ability of molecules, zeolites have been extensively applied as adsorbents, ion-exchangers and catalysts in the industry [5]. And thanks to the three-dimensional frameworks, zeolites have been employed as heterogeneous catalysts in the MTP reaction in many papers [1,3], and the high silica H-ZSM-5 zeolite was recognized as the most suitable catalyst for the MTP process [6]. The topology of MFI (ZSM-5) is also a three-dimensional framework consisting of two 10 ring channels, where the straight channels (5.1–5.5 Å) are cross-connected with the zigzag channels (5.3–5.6 Å) [7].

Although various patents have focused their work on the higher selectivity of propylene [8–10], the selectivity of propylene and the propylene/ethylene (P/E) ratio in the MTP process catalysed by conventional ZSM-5 zeolites were still very low. And among the many factors, the properties of zeolite catalysts (channel structure, acidity and crystal size) have been believed to greatly influence the catalytic performance [11]. However, due to the small and even partial occlusive micropores (less than 2 nm), the inefficient transportation of large molecules in crystals has been recognized as the most serious issue which has greatly limited the application of zeolites in the MTP reaction [12]. Thus, it is of extreme importance to modify and develop an efficient ZSM-5 zeolite catalyst in the MTP process, where an outstanding lifetime of catalysts and superior conversion of methanol and great selectivity of propylene can be obtained [13].

In spite of the ordered mesoporous materials developed [14] to improve the issue of inefficient mass transportation [15], the structural nature of amorphous materials resulted in poor hydrothermal stability, and due to the lack of surface acidity, these ordered mesoporous materials which were directly employed as catalysts during catalytic reactions, also exhibited ineligible catalytic ability [16]. Therefore, it might be difficult to solve the problem of catalytic inability by the method of developing ordered mesoporous materials to settle the diffusion limitations [17]. On account of the various advantages offered by the frameworks of zeolite, the nanosized zeolites with short intracrystalline path length have been synthesized to promote the catalytic performance, however, the nanosized single particles (approx. 20–40 nm) made these catalysts very difficult to reuse after reactions [18], and the hydrothermal stability of the nanosized zeolites was also worthy of discussion [19]. During the catalytic reactions, the coke deposition was considered as the culprit for the rapid inactivation of zeolite catalysts, and it was proved that the deposition of coke tended to occur in mesopores rather than in micropores in catalysis applications [20]. Thus, the hierarchical zeolites, which were synthesized from the introduction of mesopores (2–50 nm) into the microporous bulky domains, could efficiently improve the catalytic ability of zeolites [21,22]. These hierarchical zeolites not only had the advantages of traditional zeolites (superior surface acidity and outstanding hydrothermal stability), but also the advantages of mesoporous materials (largely external surface area and decreased diffusion path length) [23–25].

The chemical etching of the traditional zeolites, which commonly included dealumination and desilication, have been regarded as an effective method to synthesize hierarchical zeolites, whereas the mesopores etched by dealumination were always intercrystalline, which had little improvement compared to intracrystalline diffusion [26,27], and the desilication commonly resulted in the decline of the crystallinity and hydrothermal stability of zeolites [28–30], due to the stripping of silicon atoms from the frameworks and the destruction of zeolite structure [22].

The templating approaches have also been used to synthesize hierarchical zeolites with either intercrystalline or intracrystalline mesopores. In comparison to the chemical etching method, the templating approaches, including hard and soft templates, could effectively avoid the destruction of the framework properties of zeolites to a large extent [31,32]. Vast numbers of hard templates (activated carbon [33,34], aerogels [35], polymer aerogel [36] and sucrose [37]); and soft templates (cationic polymers, amphiphilic organosilane surfactants and silylated polymers) [19,23,25,38] have been tested and developed to synthesize various hierarchical zeolites. However, based on the nature of hydrophobicity of carbon, one of the drawbacks of the mesopores templated by hard templates should be the too large mesopore size and too broad pore size distribution, and it seemed to be difficult to elaborate tailoring the mesoporosity with carbon templates [39]. By reason of the actual covalent connection or charge compensation, the soft templates would react more deeply with the zeolite precursors, rather than a simple physical mix. The hierarchical zeolites with great catalytic performance have been synthesized using cationic polymers by Xiao *et al*. [40]. Choi *et al*. [41–43] have successfully produced hierarchical zeolites with uniform mesopores by using a rationally designed amphiphilic organosilane surfactant as mesoporogen. Wang & Pinnavaia [44] have synthesized hierarchical ZSM-5 zeolites of small intracrystalline mesopores by employing a silane-functionalized polymer. At the same time, because of the unsteady connection mode between templates and frameworks, these mesoporogens also had the possibility to be repelled from the zeolite precursors during the crystallization process, and finally obtained the hybrid materials of conventional zeolites and amorphous phases [45].

In short, it was necessary that an effective template should not only have hydrophobic groups to provide convenience for the formation of mesopores, but also should have a steady connection mode with the zeolite precursors during the process of crystallization [41]. The as-produced OPA template in this work has been designed to have two ternary ammonium groups in the centre which were connected with organic alkoxy chain, and each ternary ammonium group possessed two organoalkoxysilane chains which each had one silicon atom distributed terminally, and three methoxy-groups (–OCH_3_) were connected to the each silicon atoms. The OPA molecules could subsequently connect with the MFI frameworks by the covalent bonds of –Si–O–Si–, and then the OPA would be a stable phase of the zeolite precursors during crystallization [46]. The long alkyl chains of OPA played an important role in preventing the further development of crystals, forming the primary nanosized crystal domains with intra- /intercrystalline mesopores.

The main purpose of this work is to also develop a novel and efficient mesoporogen for the synthesis of hierarchical ZSM-5 zeolites with great microporosity and superior mesoporosity and excellent catalytic performance in the methanol to propylene (MTP) reaction.

## 2. Material and methods

### 2.1. Fabrication and verification of organosiloxane-polyether amine


2.1
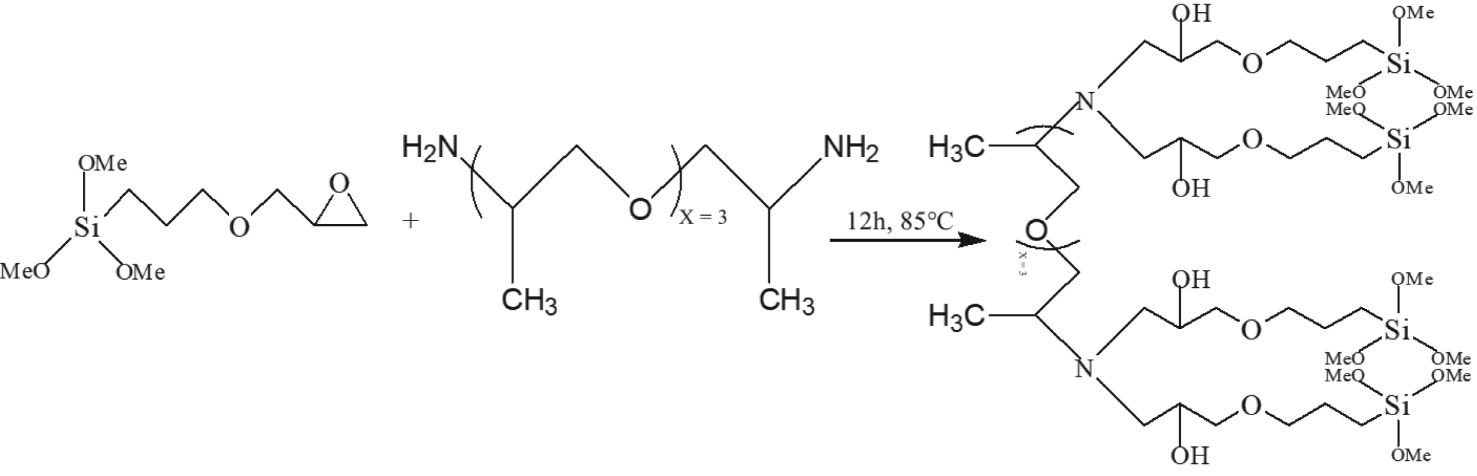
Equation (2.1). Fabrication method of OPA.

The OPA was fabricated through the method of reaction by amino-terminated polyoxypropylene (D230, C_11_H_24_N_2_O_3_, 232, Zhangjiagang Shinehoo Chemical Co. Ltd) with (3-glycidoxypropyl) trimethoxysilane (KH560, C_9_H_20_O_5_Si, 236, Qufu Yi Shun Chemical Co. Ltd) as illustrated in equation (2.1). The raw materials were stirred vigorously for 15 min, and then reacted in a microwave chemical reactor with nitrogen protection at 85°C for 12 h to obtain the OPA product. The OPA was simultaneously preserved in hermetic vials. The FTIR spectrum (electronic supplementary material, figure S1) and NMR analysis (electronic supplementary material, figure S2 and S3) were used to verify and confirm the molecular structure of OPA.

### 2.2. Synthesis of the hierarchical ZSM-5 zeolites

In the typical synthesis method of hierarchical ZSM-5 zeolites, the mesoporogen OPA was added to the solution of 22.11 g silica sol (40 wt% SiO_2_, Guangdong Huihe Silicon Products Co., Ltd) (or 30.7 g tetraethylorthosilicate (TEOS), Shanghai Silicon Mountain Macromolecular Materials Co., Ltd) and 45–68 ml tetrapropylammonium hydroxide (TPAOH, 25 wt%, C_12_H_29_NO, 203.37, Zhengzhou Alpha Chemical Co., Ltd), after further rapid stirring for 2 h, the obtained emulsion was named as A. Sodium aluminate of 0.11 g (NaAlO_2_, 82, Shanghai Kaiyun Medical Technology Co., Ltd) was added into 25 ml distilled water for 10–15 min with rapid stirring, and the obtained solution was named as B. Solution A was then added into solution B, and the precursor was further stirred for 2 h. The precursor was transferred into Teflon coated stainless-steel autoclaves and hydrothermally crystallized at 80°C for 24 h and then 140–180°C for 1–7 days. Lastly, the samples were washed and filtrated, and dried at 110°C for 12 h and then calcined in air at 550°C for 10 h. The typical molar composition of the precursor was 220 SiO_2_: Al_2_O_3_: 82.6–124.8 TPAOH: 5974–7404 H_2_O: 2–7 OPA. The optimal synthesis conditions with silica sol and TEOS as silicon sources were 160°C for 5 days and 160°C for 6 days, respectively. The samples produced with silica sol were named as SMZ, and the samples obtained with TEOS were named as TMZ. The conventional microporous ZSM-5 zeolite, named CZ, was synthesized under the same method of SMZ in the absence of OPA. The Na-form samples were exchanged for 0.5 mol l^−1^ NH_4_NO_3_ at 90°C for 1 h with a solution/sample ratio of 10 cm^3^ g^−1^, repeated three times, followed by calcination at 550°C for 6 h to obtain H-form samples.

### 2.3. Characterization

Powder X-ray diffraction (XRD) analysis was carried out in a Shimadzu XRD-6000 diffractometer equipped with a copper tube (*λ* = 0.15418 nm). Nitrogen sorption analysis was performed on a Quantachrome Nova 2000e Surface Area & Pore Size Analyzer. Prior to analysis, all the samples were degassed at 300°C for 10 h. The t-plot method was employed to estimate the micropore volume, and micropore surface area and external surface area. The density functional theory (DFT) method was applied to assess the mesopore size distribution. Scanning electron microscopy (SEM) images were obtained on a Hitachi S4800 instrument at 5 kV. Transmission electron microscopy (TEM) images were obtained on a Philips FEI Tecnai G2 F20 microscope at 200 kV. The surface acidity was operated by NH_3_ temperature-programmed desorption (NH_3_-TPD) on a Finetec Finesorb 3010 analyzer. The thermogravimetric (TG) measurement was conducted on a Netzsch Sta 449 F3 instrument. The SiO_2_/Al_2_O_3_ molar ratio was measured through inductively coupled plasma on a Varian 720 instrument.

### 2.4. Catalytic reaction

Before testing, the catalyst powder was pretreated at 5 tons for 10 min, crushed and sieved to 40–60 mesh particles. The MTP reaction was conducted at 470°C with the pressure of 1 atm in a fixed bed reactor of 24 mm diameter. The amount of catalysts used was 1.0 g. Before each reaction, the catalysts should be activated at 470°C for 1.5 h under a flow of highly purified N_2_ (99.999%, 45 ml min^−1^). The reactant of methanol was mixed with distilled water of the same molar ratio and the mixture was pumped with a metering pump. The weight hourly space velocity of methanol was 6 h^−1^. All products were characterized on a Varian CP3800 gas chromatograph with an FID detector. The conversion of methanol and the selectivity of products were calculated through equations (2.2) and (2.3).

2.2 $$\mathrm{Methanol}\;\mathrm{conversion}\;(\text{\%})\;=\;\frac{N_{\mathrm{MeOH}}^{i}-(N_{\mathrm{MeOH}}^{o}+2N_{\mathrm{DME}}^{o})}{N_{\mathrm{MeOH}}^{i}}\times 100,$$

2.3 $$\;\mathrm{and}\;\mathrm{Selectivity}\;(\text{\%})=\frac{X\times N_{C_{x}H_{y}}^{o}}{N_{\mathrm{MeOH}}^{i}-(N_{\mathrm{MeOH}}^{o}+2N_{\mathrm{DME}}^{o})}\times 100.$$

*N* represented the number of moles. Superscripts *i* and *o* were the components at the inlet and outlet of the reactor. The value *x* was the number of carbon atoms and *y* was the number of hydrogen atoms. The Dimethyl ether was considered as an unconverted reactant.

## 3. Results and discussion

The pore parameters of the SMZ samples synthesized in the presence of different amounts of OPA were presented in table 1, from which the sample CZ was also characterized for comparison. The hierarchical factor was calculated to investigate the influence of mesoporogen on the porosity of hierarchical zeolites [47]. Although the hierarchical factor of the SMZ samples was slightly lower than that of the CZ samples (0.11), the SMZ samples in this work had larger external surface area (345–463 m^2^ g^−1^) which was superior to that of the reported works [48–55], while the micropore surface area could still maintain high levels (307–334 m^2^ g^−1^). The decrease of HF in SMZ samples might be ascribed to the larger meosopore size introduced by OPA, which resulted in the increase of total pore volume [56].

**Table 1.** Pore properties of CZ and SMZ samples obtained at 160°C for 5 days.

samples	*S*_BET_^a^ (m^2^ g^−1^)	*S*_mic_^b^ (m^2^ g^−1^)	*S*_ext_^c^ (m^2^ g^−1^)	*V*_mic_^d^ (cm^3^ g^−1^)	*V*_total_^e^ (cm^3^ g^−1^)	OPA amounts (ml)	HF^f^
CZ	384	330	54	0.14	0.18	0	0.11
SMZ-1	654	309	345	0.13	1.2	6	0.06
SMZ-2	744	334	410	0.14	0.9	8	0.09
SMZ-3	795	332	463	0.14	0.8	10	0.10
SMZ-4	777	332	445	0.13	0.8	12	0.09

^a^BET surface area.

^b^Micropore surface area.

^c^External surface area.

^d^Micropore volume.

^e^Total pore volume.

^f^The hierarchical factor, defined as (*V*_mic_/*V*_total_) × (*S*_ext_/*S*_BET_).

**Figure 1.**
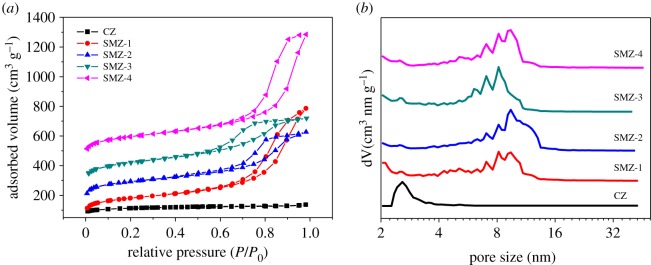
(*a*) N_2_ sorption isotherms and (*b*) corresponding mesopore size distributions of SMZ samples and the sample of CZ.

**Figure 2.**
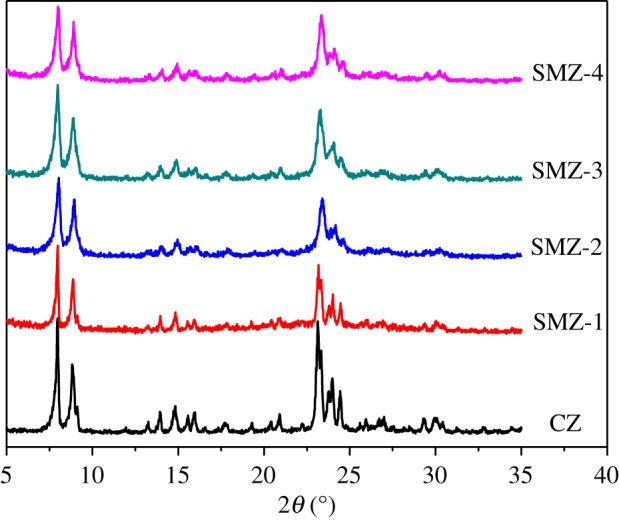
XRD patterns of SMZ samples and the sample of CZ.

Figure 1 showed the N_2_ sorption isotherms (A) and the corresponding mesopore size distributions (B) of SMZ samples. It was obvious that all isotherms exhibited a hysteresis loop attributed to the capillary condensation of N_2_ in mesopores [56]. However, the hysteresis loops of SMZ-1 and SMZ-4 were of type H3, which resulted from the slit mesopores; and the hysteresis loops of SMZ-2 and SMZ-3 were of type H2, indicating the formation of inkpot-like mesopores. The mesopore size distributions of SMZ samples centered around 7–15 nm, which was in stark contrast with that of CZ. Figure 2 displayed the XRD patterns, and all the SMZ samples possessed the same diffraction peaks as the CZ samples, and no amorphous or other impure phases were found, implying the pure MFI frameworks of the SMZ samples. In addition, it was reasonable that the diffraction intensities had decreased to some extent due to the vast introduction of mesopores and the formation of nanocrystal domains [57]. The SMZ samples in table 1 were obtained under the same optimal synthesis condition, and it was found that the mesoporogen OPA had an influence on the types of mesopores, perhaps as a result of the complicated molecular structure of OPA and the uncontrollable blocking process. However, the OPA had little impact on the microposity of these SMZ samples, further demonstrating the great stability of the connection mode between OPA and zeolite precursors in the way of covalent bonds (–Si–O–Si–) which formed from the reaction of hydrolysed alkoxane of OPA and the silicon hydroxyl (OH–Si–) of silicon sources.

**Figure 3.**
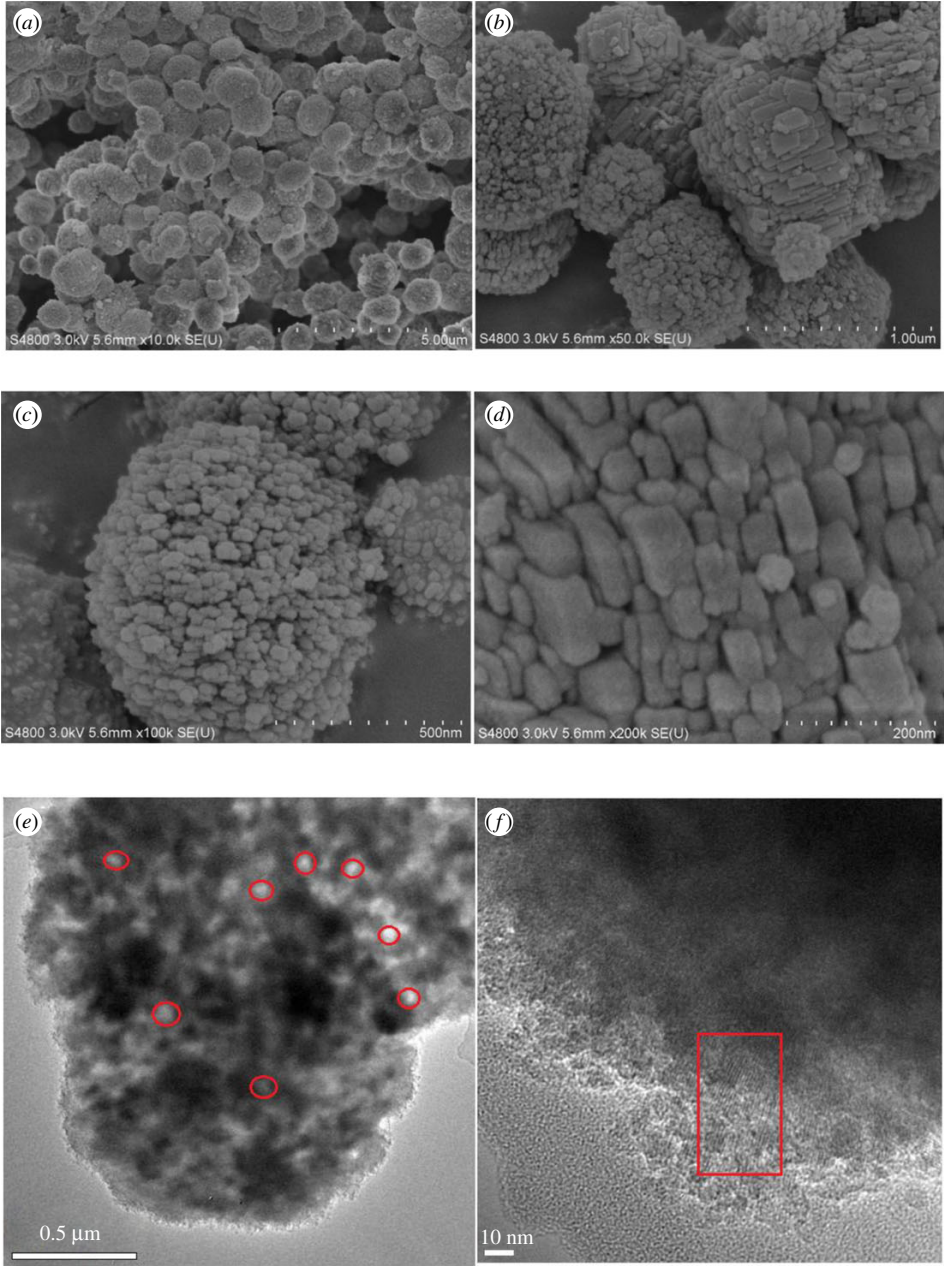
(*a–d*) SEM images and (*e–f*) TEM images of SMZ-3.

The SEM images of sample SMZ-3 were displayed in figure 3, and it was obvious that the particles were quasi-circular in sizes of about 800 nm which consisted of nanocrystals of 40 nm. It was certain that this morphology resulted from the formation of abundant mesopores in zeolites [57], and the nanocrystals and intracrystalline mesopores greatly shortened the diffusion path length and promoted the mass transportation in particles [58]. From the TEM images (E), it could be clearly deduced that intercrystalline mesopores were also formed between the nanocrystals [57] and the larger mesopore size (approx. 40 nm) was not detected in the DFT pore size distribution in figure 1*b*. This high ‘aggregation’ morphology with micro–meso–macropores was conducive to the reactants' assessing the active acid sites effectively [54] and could improve the catalytic performance of the MTP process [55]. The clear lattice fringes in (F) have also been assumed to imply the microporous zeolite structure of SMZ samples.

As shown in table 2, the pore properties of TMZ samples synthesized with TEOS as the silicon source exhibited lower values of the external surface area, micropore surface area and BET surface area than that of SMZ samples produced with silica sol as the silicon source, in the presence of the same amounts of OPA. The micropore surface area of TMZ samples decreased by about 22% compared with sample CZ, and the largest external surface area of TMZ samples was 363 m^2^ g^–1^ which decreased 100 m^2^ g^–1^ compared to sample SMZ-3. These changes should primarily be attributed to the inevitable hydrolysis procedure of TEOS to possess OH–Si–, however, it was needless for silica sol. It was easier for OPA molecules to connect with silica sol than TEOS at the initial crystallization process, and due to the relatively slower connection rate in the TEOS precursor, some OPA molecules were excluded from the formation of zeolite frameworks and retained in the mother liquor, ultimately caused the inefficient mesopore-forming ability.

**Table 2.** Pore properties of TMZ samples synthesized at 160°C for 5 days.

samples	*S*_BET_^a^ (m^2^ g)	*S*_mic_^b^ (m^2^ g^−1^)	*S*_ext_^c^ (m^2^ g^−1^)	*V*_mic_^d^ (cm^3^ g^−1^)	*V*_total_^e^ (cm^3^ g^−1^)	OPA amounts (ml)	HF^f^
TMZ-1	430	245	185	0.10	0.45	8	0.10
TMZ-2	525	257	268	0.11	0.67	10	0.08
TMZ-3	622	259	363	0.10	0.63	12	0.09
TMZ-4	549	246	303	0.12	0.68	14	0.10

^a^BET surface area.

^b^Micropore surface area.

^c^External surface area.

^d^Micropore volume.

^e^Total pore volume.

^f^The hierarchical factor, defined as (*V*_mic_/*V*_total_) × (*S*_ext_/*S*_BET_).

**Figure 4.**
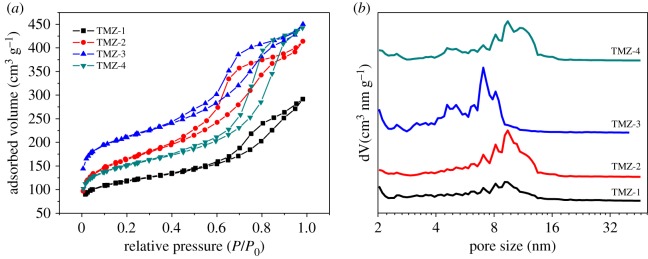
(*a*) N_2_ sorption isotherms and (*b*) corresponding mesopore size distributions of TMZ samples.

**Figure 5.**
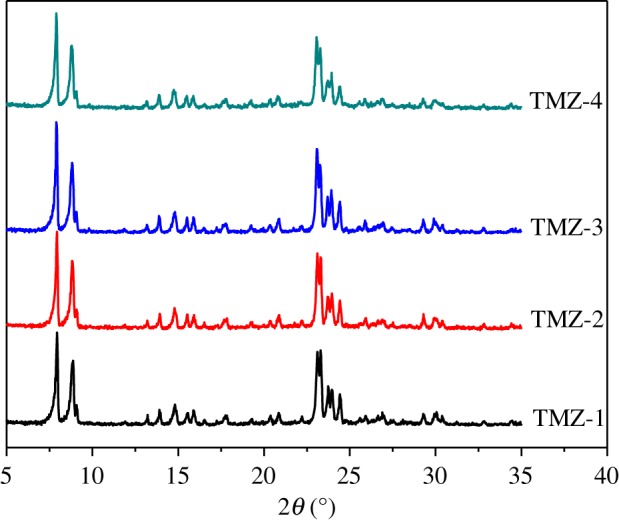
XRD patterns of TMZ samples.

The N_2_ sorption isotherms of TMZ samples in figure 4*a* exhibited typical type VI with hysteresis loop of H3. The corresponding mesopore size distribution of sample TMZ-3, around 6–10 nm in (B), was narrower than that of the other TMZ samples of 6–15 nm. It could be speculated that a delicate balance existed between the amounts of OPA and the PH of synthesis mixture, and because the hydrolysis of OPA might result in subtle changes of PH, the poly-reaction among OPA molecules happened to some extent, which resulted in wider pore size distribution. The XRD patterns in figure 5 demonstrated the pure MFI structure of TMZ samples, although with a decrease in microporosity. Therefore, it was also practicable to synthesize hierarchical ZSM-5 zeolites with TEOS as silicon source in the presence of OPA.

**Figure 6.**
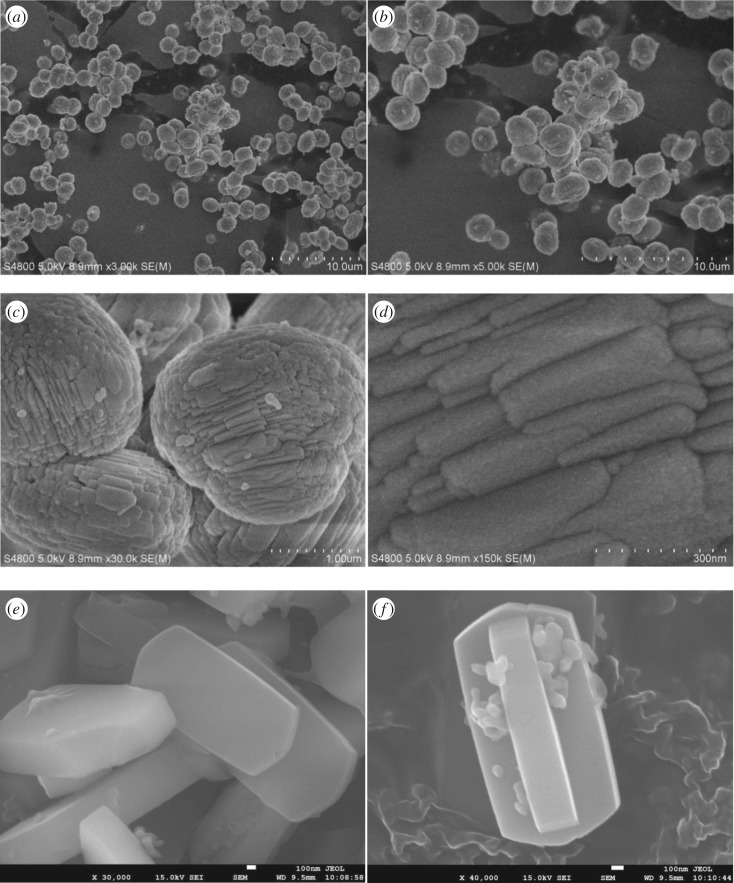
(*a–d*) SEM images of TMZ-3 and (*e–f*) CZ.

**Figure 7.**
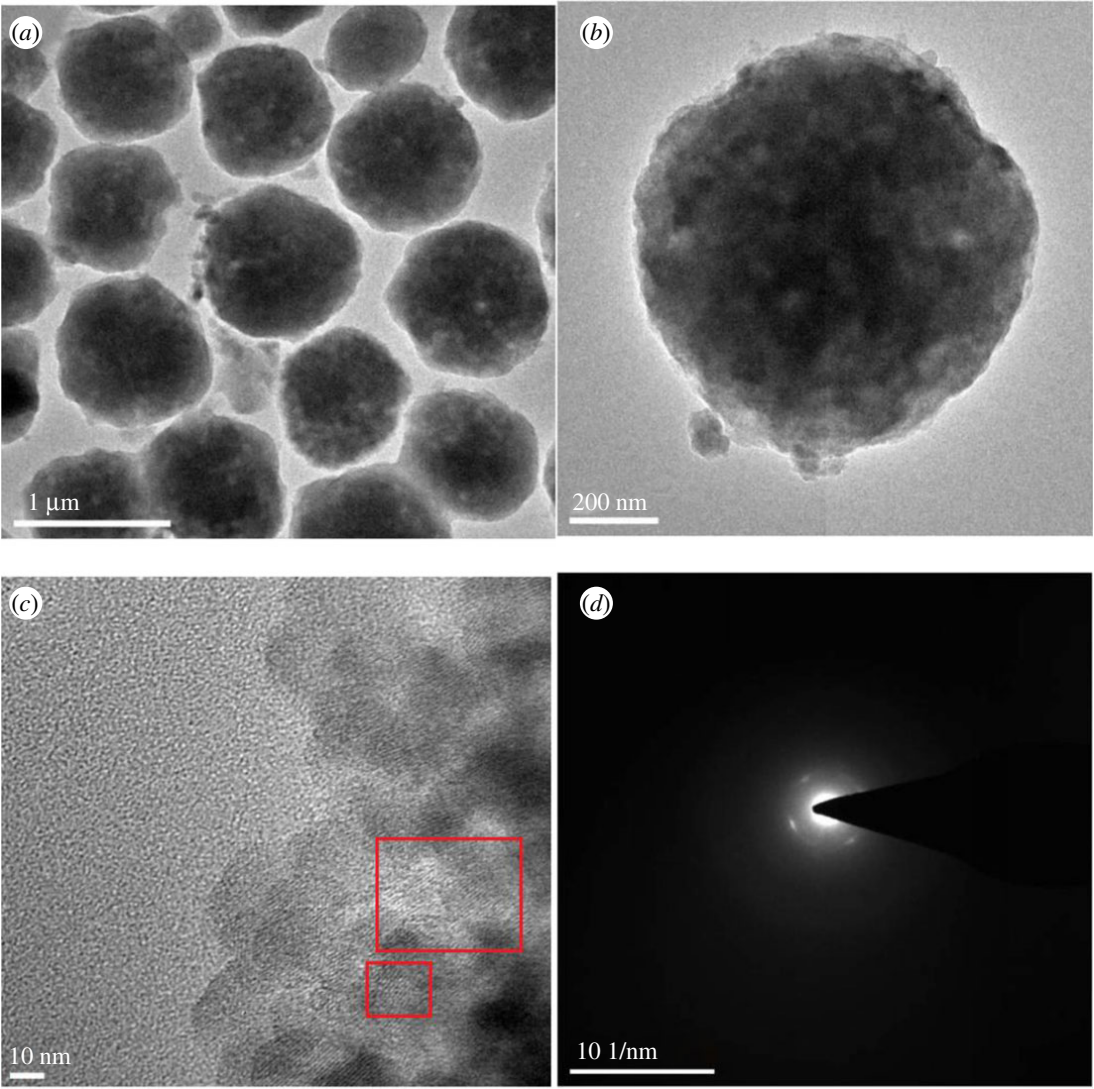
TEM images of TMZ-3.

The SEM images of TMZ-3 in figure 6*a–d* presented spheroid particles in sizes of about 1–2 µm, which were further aggregated with nanosheets of about 300 nm, and these particles were more compact than the particles of SMZ-3, which were also different from the images of CZ shown in figure 6*e–f* that were of coffin-like morphology in sizes of 2–5 µm. And the corresponding TEM images were showed in figure 7, from which very few intermesopores have been found. The difference of particle morphology between SMZ-3 and TMZ-3 probably originated from the different initial connection rates of OPA with the different silicon sources, and it seemed that compact nano-aggregates were formed by reason of the exclusion of OPA molecules from the nanosheets. The lattice fringes of microporous zeolite framework have been displayed in figure 7*c* and the selected area electron diffraction was also employed to verify the single crystal structure.

**Figure 8.**
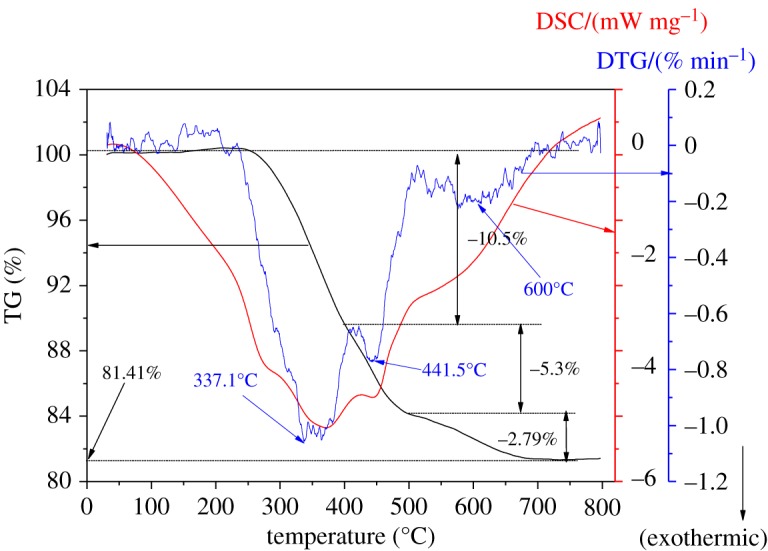
TG-DSC/DTG curve of SMZ-3.

As shown in figure 8, the sample of SMZ-3 was characterized by TG-DSC/DTG techniques to demonstrate the existence of OPA and to investigate the combustion temperature of the different organic moieties. The weight loss of the traditional uncalcined ZSM-5 sample could be classified into three regions at 50–350°C, 350–550°C and 550–800°C as shown in electronic supplementary material, figure S4, attributing to the removal of H_2_O of 2.0%, burning of TPA^+^ of 7.2%, and combustion of TPA^+^ occluded in occlusive cages of 2.7%, respectively [59]. The DSC and DTG curves in electronic supplementary material, figure S8 almost showed the same two peaks at around 460°C and 600°C, corresponding with the decomposition of TPA^+^ [60]. From the thermal gravimetric analysis in electronic supplementary material, figure S8, the TG curve of SMZ-3 was divided into three steps: at first, 10.5% between 250°C and 400°C should be ascribed to the combustion of the mesoporogen OPA; 5.23% between 400°C and 500°C and then 2.79% at 500–800°C, all corresponding to decomposition of TPA^+^ located in various cages. Moreover, it should be pointed that there was a peak of the DSC curve located at 375°C, ascribed to the combustion of the hydrocarbon moiety and the tertiary amine moiety of OPA.

**Figure 9.**
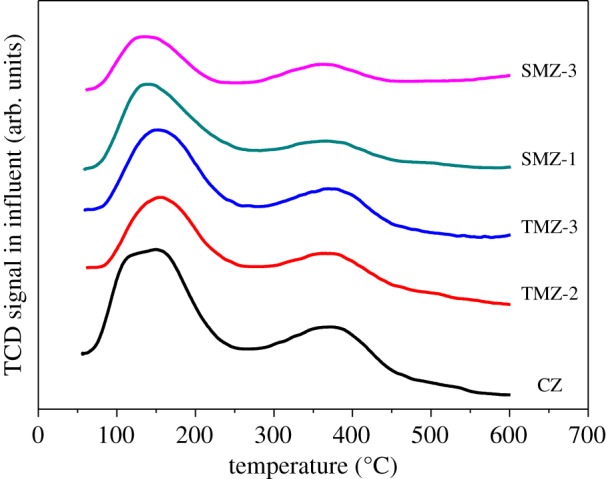
NH_3_-TPD curves of the H-form SMZ and TMZ samples and the sample of CZ.

As shown in figure 9, the NH_3_-TPD curves of the H-form hierarchical ZSM-5 zeolites all exhibited two typical peaks at 150°C and 400°C, belonging to the weak and strong acid sites, respectively [27]. The peak located around the low-temperature region could be ascribed to the interaction between the hydrogen bond and silicon-oxygen bond, and the peak around the high-temperature position was relevant to the framework aluminium [61,62]. Although these samples had the same SiO_2_/Al_2_O_3_ molar ratios (SiO_2_/Al_2_O_3_ = 200) as CZ, the nanosized primary crystals of the SMZ and TMZ samples caused a slight decrease in the amounts of the weak or strong acid sites [54]. And as to the strong acid sites, the SMZ samples showed lower amounts than the TMZ samples, which might be ascribed to the smaller nanocrystals domains of SMZ, indicating that the nanosized extent of hierarchical samples had a direct impact on the strong acid sites.

**Figure 10.**
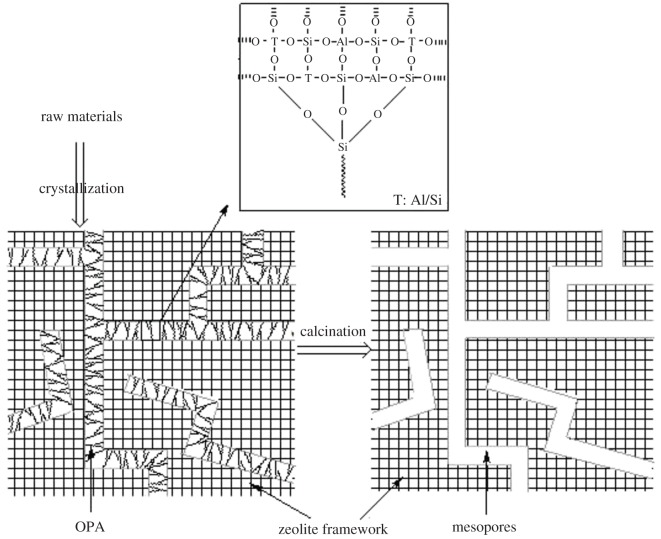
The mechanism of the synthesis of hierarchical ZSM-5 zeolites in the presence of OPA.

The mechanism of the synthesis of hierarchical ZSM-5 zeolites in the presence of OPA has been proposed in figure 10, where the connection mode between OPA molecules and MFI framework was illustrated. After the hydrolysis of methoxy, the groups of –Si–OH in OPA molecules reacted with the groups of –Si–OH distributed over appearance of zeolite precursors in condensation. According to the rules of atom coordination in zeolite frameworks, Al atoms could not connect with Al to form –Al–O–Al–, and Si atoms could connect with either Al or Si atoms to form –Si–O–Al– or –Si–O–Si–, and therefore, the covalent bonds of –Si–O–Si– formed as shown in the inset of figure 10. Meanwhile, as a stable fraction, the OPA molecules experienced the high-temperature crystallization process with zeolite precursors. After crystallization, the mesoporogen OPA could be removed from the frameworks of zeolites by calcination and the mesopore channels were uniformly distributed in crystals.

**Figure 11.**
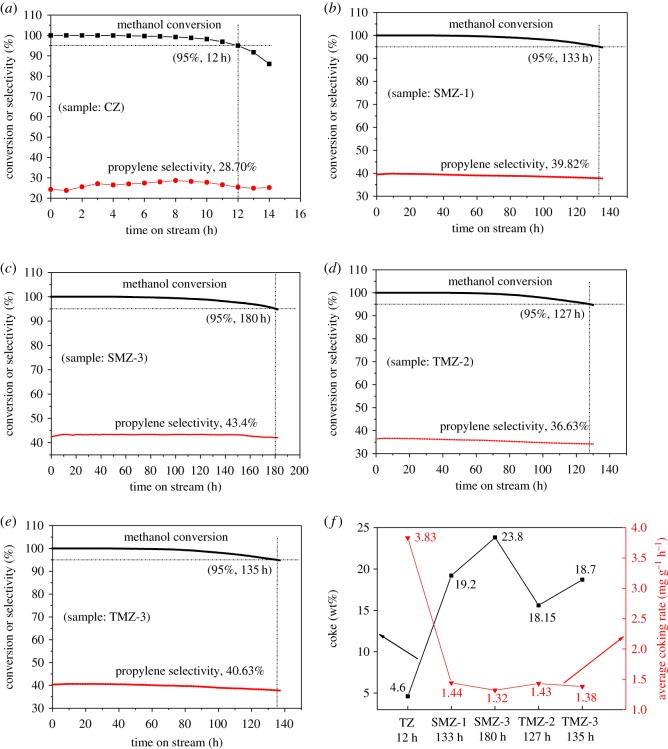
The methanol conversion and selectivity of propylene of the hierarchical ZSM zeolites in MTP reaction.

The MTP reaction was employed to assess the catalytic activity of the hierarchical ZSM-5 samples synthesized in this work. The reaction was stopped after the conversion of methanol dropped to 95%, and the reaction time was used to characterize the carbon resistance ability of catalysts, while the selectivity of propylene could be used to investigate the suitability of catalysts in the MTP reaction. The detailed selectivity of the other products was displayed in table S1. As shown in figure 11*a*, after reacting for 12 h on sample CZ, the conversion of methanol dropped to 95% and the selectivity of propylene was only 28.7%. The reaction times of the SMZ-1 and SMZ-3 samples were 133 h and 180 h, and the selectivity of propylene was 39.82% and 43.4%, respectively. The longer reaction time of SMZ-3 should be attributed to the larger external surface area and the more intra-/intercrystalline mesopores compared to SMZ-1, which provided convenience for the diffusion of reactants and products to promote the carbon resistance ability of catalysts. And although the total coke amount of SMZ-3 reached 23.8 wt%, the average coking rate was only 1.32, which was the minimum as calculated in figure 11*f*. The reaction times of the samples of TMZ-2 and TMZ-3 were 127 h and 135 h, which were significantly longer than the results in the reported works [54,55] but shorter than that of SMZ-3; and the selectivity of propylene was 36.63% and 40.63%, respectively, further indicating that the hierarchical ZSM-5 zeolites synthesized either in silica sol or TEOS had great catalytic performance in the MTP reaction. Therefore, in comparison to the high average coking rate of sample CZ, the hierarchical zeolites exhibited much lower rates from 1.32 to 1.44, benefiting from the outstanding mesoporosity.

## 4. Conclusion

A novel molecule (OPA) was successfully fabricated with a kind of organosiloxane and polyetheramine using a simple method, and the OPA was further employed as a mesoporogen to synthesize hierarchical ZSM-5 zeolites in the presence of different silicon sources. The intracrystalline mesopores in sizes of 6–15 nm could be detected both in the SMZ and the TMZ samples, however, only in the SMZ samples were the plentiful intercrystalline mesopores of about 40 nm characterized. The amounts of OPA could slightly affect the PH of the synthesis mixture, which gave rise to the change of mesopore types and resulted in poly-reaction among OPA molecules to some extent. The synthesis conditions should be optimized elaborately due to the complicated molecular structure of OPA. The samples produced under optimal conditions exhibited great microporosity and superior mesoporosity as well as similar acidity. These samples were then used as catalysts in the MTP reaction, and the reaction time was greatly promoted and the selectivity of propylene could be maintained at high levels. It was proved that the structure of OPA could connect with the zeolite precursor steadily and avoid the formation of amorphous or other phases. Not only could the hierarchical ZSM-5 zeolites be obtained with this method, but also the other types of zeolites could be produced, and the other types of mesoporogen could be synthesized according to the fabrication principle of OPA.

## Supplementary Material

The FTIR spectrum of OPA.; The 1H NMR of OPA.; The 13C NMR of OPA.; TG-DSC/DTG curve of the uncalcined traditional ZSM-5 sample.

## Data accessibility

This article does not contain any additional data.
