# Supplementary material for: Synthesis of an excellent MTP catalyst: hierarchical ZSM-5 zeolites with great mesoporosity
Source: R Soc Open Sci. 2018 Dec 12;5(12):181691. doi: 10.1098/rsos.181691 (PMC6304151; doi:10.1098/rsos.181691)
Supplement: The FTIR spectrum of OPA.; The 1H NMR of OPA.; The 13C NMR of OPA.; TG-DSC/DTG curve of the uncalcined traditional ZSM-5 sample. [file rsos181691supp1.docx]

**<Supplementary Information>**

**Synthesis of an excellent MTP catalyst: Hierarchical ZSM-5 zeolites with great mesoporosity**

Guoqiang Song ^a,b,c^, Wenting Chen ^a^, Peipei Dang ^a^, Yuanyi Wang ^a^, Fuxiang Li ^d,^*

^a^ School of chemical engineering, Guizhou Institute of Technology, 1st Caiguan Road, Yunyan District, Guiyang City 550003, Guizhou Province.

^b^ 2011 Special Functional Materials Collaborative Innovation Center of Guizhou Province, Guizhou Institute of Technology.

^c^ Key Laboratory of Light Metal Materials Processing Technology of Guizhou Province, Guizhou Institute of Technology.

^d^ College of chemistry and chemical engineering, Taiyuan University of Technology, No. 79 Yingze west street, Taiyuan City 030024, Shanxi Province.

* Email addresses of corresponding author: [l63f64x@163.com](mailto:L63f64x@163.com);

Tel. : (+86) 0351-6111178.

Fig. 1S The FTIR spectrum of OPA.

The FTIR spectrum of OPA showed the disappearances of the absorption bands at 900 cm^-1^ and 3500-3300 cm^-1^, which could be assigned to the [epoxy group](http://www.iciba.com/epoxy_group)s in the (3-glycidoxypropyl) trimethoxysilane and the amino groups in the amino-terminated polyoxypropylene (D230, C_11_H_24_N_2_O_3_, 232), respectively. This FTIR result confirmed that the epoxide ring open to form hydrolytically stable C-N bonds with the amino groups showed in Equation. 1, and consequently obtained the OPA product.
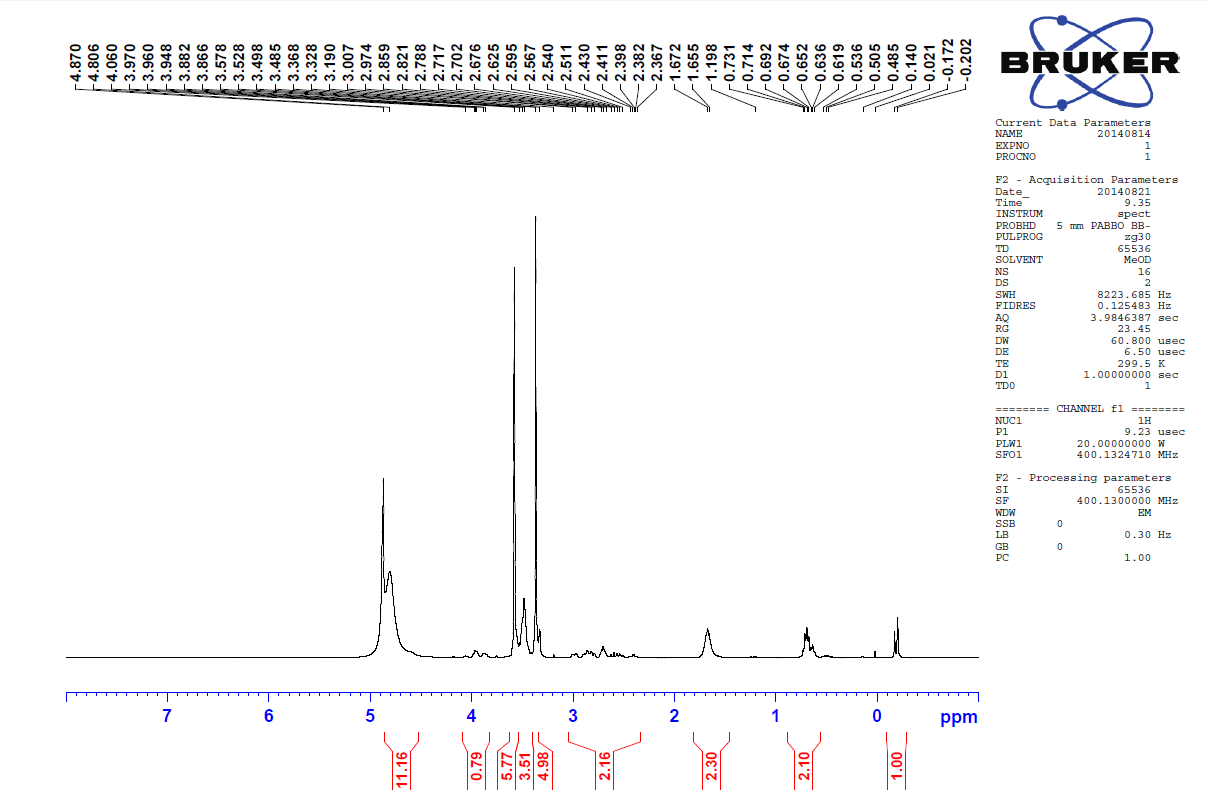


Fig. 2S The ^1^H NMR of OPA.

The ^1^H NMR spectrum is performed to characterize the structure of OPA. The spectra exhibits main resonances at 3.3-3.8 ppm and 4.5-5.0 ppm, verifying the presence of H-C-O (CH_3_-O-, CH_2_-O-, and CH-O-) and H-O-C (HO-CH-, attributed to the epoxide ring open), respectively. Moreover, three distinguishable resonances at 0.5-0.9 ppm, 1.5-1.9 ppm and 2.2-2.9 ppm appear in the ^1^H NMR spectrum, which can be assigned to (H-C-C), (H-C-Si) and (H-C-N-R) respectively.
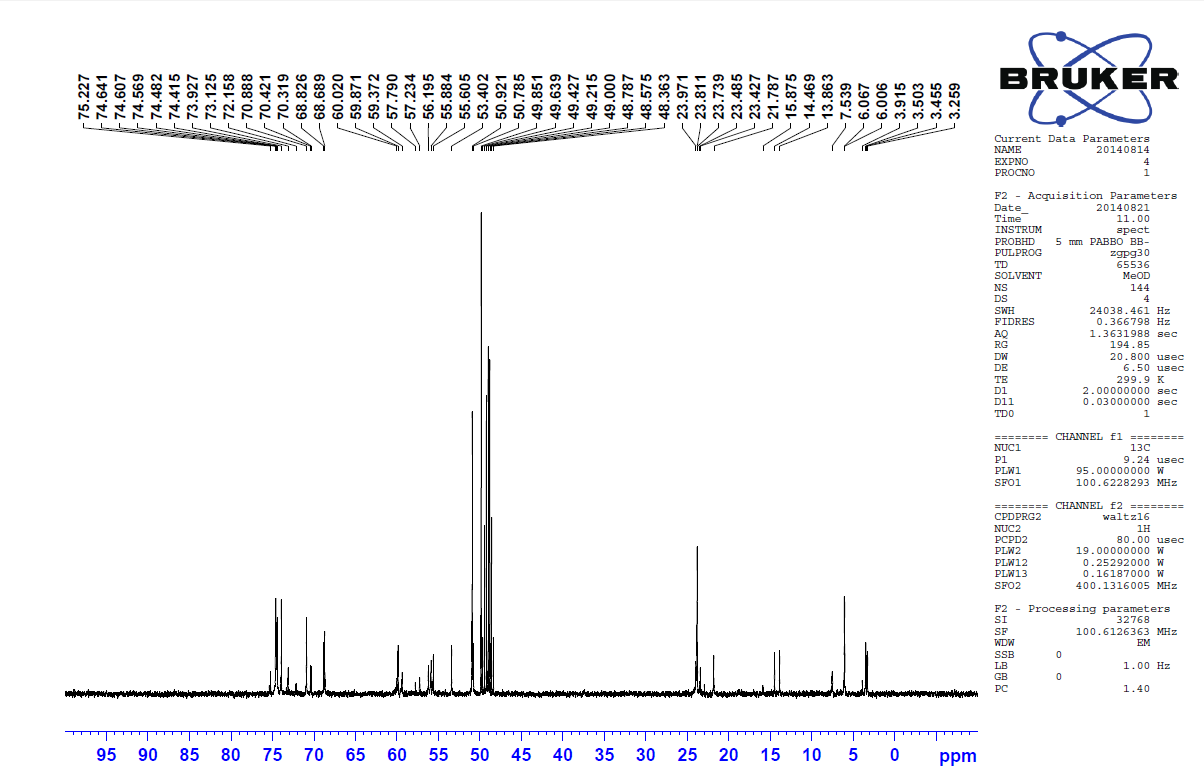


Fig. 3S The ^13^C NMR of OPA.

The ^13^C NMR spectrum is also employed to characterize the structure of OPA. The spectra presents main resonances at 13-25 ppm, 47-60 ppm, and 67-77 ppm, indicating respectively the presence of (C-CH_2_-), (R-C-O- and R-C-N-) and (C-O-). These results from FTIR and NMR analysis further demonstrate that the structure of OPA as-synthesized was coincided with the Equation. 1.

Fig. 4S. TG-DSC/DTG curve of the uncalcined traditional ZSM-5 sample.

Table. 1S Catalytic performances of MTP reaction over the hierarchical ZSM-5 catalysts.

| Catalysts | Lifetime / h | Selectivity / % | | | | | | | | | | P/E |
| --- | --- | --- | --- | --- | --- | --- | --- | --- | --- | --- | --- | --- |
|  |  | CH_4_ | C_2_H_6_ | C_2_H_4_ | C_3_H_6_ | C_4_H_12_ | C_4_H_8_ | C_5_H_10_ | C_6_H_12_ | C_7_H_14_ | Aromatics *^a^* |  |
| CZ | 12 | 1.02 | 0.04 | 9.93 | 28.7 | 5.48 | 16.12 | 18.53 | 7.54 | 3.13 | 9.51 | 2.9 |
| SMZ-1 | 133 | 1.45 | 0.07 | 5.17 | 39.82 | 7.04 | 18.75 | 13.44 | 7.42 | 1.52 | 5.32 | 7.7 |
| SMZ-3 | 180 | 1.77 | 0.09 | 4.89 | 43.4 | 6.58 | 19.79 | 10.14 | 7.04 | 1.28 | 5.02 | 8.9 |
| TMZ-2 | 127 | 1.34 | 0.07 | 6.45 | 36.63 | 6.95 | 18.16 | 15.07 | 8.23 | 1.40 | 5.70 | 5.7 |
| TMZ-3 | 135 | 1.64 | 0.08 | 5.35 | 40.63 | 6.55 | 18.41 | 14.02 | 7.34 | 1.02 | 4.96 | 7.6 |

^a^ Aromatics include benzene, toluene, xylene and higher aromatics.
